# Respiratory toxicity of persulphate salts and their adverse effects on airways in hairdressers: a systematic review

**DOI:** 10.1007/s00420-022-01852-w

**Published:** 2022-03-22

**Authors:** Jelena Macan, Željka Babić, Sarah Hallmann, Martin S. Havmose, Jeanne D. Johansen, Swen M. John, Marija Macan, Cara Symanzik, Wolfgang Uter, Patricia Weinert, Henk F. van der Molen, Sanja Kezic, Rajka Turk

**Affiliations:** 1 Institute for Medical Research and Occupational Health, Ksaverska cesta 2, 10000 Zagreb, Croatia; 2 Department of Medical Informatics, Biometry and Epidemiology, University of Erlangen, Erlangen, Germany; 3 National Allergy Research Centre, Department of Skin and Allergy, University of Copenhagen, Gentofte Hospital, Copenhagen, Denmark; 4 Department of Dermatology, Environmental Medicine and Health Theory, Osnabrück University, Osnabrück, Germany; 5 Institute for Interdisciplinary Dermatological Prevention and Rehabilitation (iDerm), Osnabrück University, Osnabrück, Germany; 6 Department of Public and Occupational Health, Coronel Institute of Occupational Health, Amsterdam Public Health Research Institute, Amsterdam UMC, University of Amsterdam, Amsterdam, The Netherlands

**Keywords:** Hair bleach, Occupational rhinitis, Occupational asthma, Hairdressing apprentices, Lung function, Specific inhalatory challenge

## Abstract

**Objective:**

To review the literature on respiratory effects of persulfate salts (PS) or hair bleaches in hairdressers and animal models exploring mechanisms behind PS-induced asthma.

**Methods:**

A systematic review according to the PRISMA guidelines was performed. Studies published from 2000 to July 2021 that fulfilled predefined eligibility criteria were retrieved. Data were not quantitatively synthesized due to the heterogeneity of study designs, outcomes and methods.

**Results:**

Forty-two articles were included. PS are indicated as the main cause of occupational rhinitis and asthma in hairdressers, and one of the leading causes of occupational asthma in some European countries. Bleaching products are indicated as the most important factor for development of respiratory symptoms, lung function decline, and leaving the hairdressing profession. Risk estimates from a good quality prospective study showed up to 3.9 times higher risk for wheezing and breathlessness in hairdressers aged ≥ 40 years than in matched controls, and 20 times higher risk in hairdressers to develop respiratory symptoms from exposure to bleaching powder than controls. Pathophysiological mechanisms of the respiratory response to PS are not yet fully elucidated, but may include non-specific and specific immune responses.

**Conclusions:**

Hairdressing is associated with a wide spectrum of respiratory adverse effects, of which bleaching products were indicated as the most hazardous. Preventive measures for reducing inhalatory exposure to PS in hair salons should be re-evaluated, including adopting occupational exposure limits at EU level, and encouraging use of safer bleach formulations.

**PROSPERO registration number:**

CRD42021238118.

**Supplementary Information:**

The online version contains supplementary material available at 10.1007/s00420-022-01852-w.

## Introduction

Persulfate salts (PS) commonly used in hair products are ammonium ((NH_4_)_2_S_2_O_8_; CAS 7727-54-0 [APS]), potassium (K_2_S_2_O_8_; CAS 7727-21-1[PPS]) and sodium (Na_2_S_2_O_8_; CAS 7725-27-1) persulfate. These water-soluble, highly reactive low molecular compounds are used to color, lighten or bleach hair, but are also found in tonics, hair conditioners, and other hair grooming aids, and cosmetic products such as eye make-ups and toothpastes. Hairdressing products generally contain a combination of two or, sometimes, all three PS at concentration range from 0.1 to 60%, formulated as powders (mixed with hydrogen peroxide just before application), creams or liquids (Cosmetic Ingredient Review 2018).

Due to their properties as water-soluble inorganic salts, PS rapidly hydrolyze upon contact with water or water vapour to form cations (ammonium, potassium, sodium) and sulfate anions which are physiologically present in organisms. Considering their low vapour pressures, exposure to PS via inhalation is unlikely unless they are aerosolized during use. Dermal absorption is likely to be neglectable as was confirmed in skin toxicity studies with primary local effects revealed. Following oral administration PS salts will hydrolyze in the acid environment of the stomach to the corresponding cations, and PS anions will undergo further decomposition to sulfate species. Consequently, they are not likely to be systemically available as PS by inhalation, ingestion, or skin exposure (Cosmetic Ingredient Review 2018).

Toxicity and adverse health effects of persulfates have been previously reviewed by several expert panels and regulatory bodies (Cosmetic Ingredient Review 2018; NICNAS 2001; DFG 2002; OECD 2005; De Wit-Bos et al. 2014; ANSES 2019) and identified as health risk in the hairdressing sector (EU-OSHA 2014). Acute toxicity studies in rats showed relatively low oral and dermal toxicity while acute inhalation exposure resulted in gross lesions of the lungs, liver, stomach, and spleen. When rats were exposed to aerosolized APS for 7 days, evidence of severe lung damage was observed. PS were tested positive in the guinea pig sensitization assay and in the mouse local lymph node assay (LLNA). No evidence of genotoxicity, reproductive/developmental toxicity, and tumor promotion or carcinogenicity was found in the available animal data.

Data about the adverse health effects of PS in humans from studies published before 2000 originate mostly from case reports showing dermal and respiratory problems associated with exposure to PS in occupational (mostly chemical industry workers and hairdressers) and in non-occupational conditions (mostly hairdressers' clients undergoing hair bleaching). Cases of irritant dermatitis, allergic contact dermatitis, localized contact urticaria, generalized urticaria, rhinitis, asthma, and syncope were reported (Pang and Fiume 2001). However, data on the prevalence and incidence of respiratory disorders caused by PS are missing. While the pathophysiological mechanism of delayed-type (type IV) allergic reaction is well documented as a basis for development of allergic contact dermatitis, data on the mechanisms underlying respiratory responses are scarce and not consistent. It was shown that PS are respiratory irritants, and can act as non-specific histamine liberators, but there was also clinical evidence about the sensitizing properties of PS by mean of immediate-type (type I) allergic reaction (DFG 2002; Pang and Fiume 2001).

The aim of this study was to review the literature published in the last 20 years regarding respiratory effects of PS or hair bleaches in hairdressers and animal models exploring mechanisms behind PS induced asthma. We searched for new data on the prevalence, incidence, risk estimates, and pathophysiological mechanisms of respiratory responses caused by PS in hairdressers as the high-risk occupation.

## Methods

This study is a part of a project reviewing toxicity of important hazardous hair and nail cosmetic ingredients in hairdressers. A detailed protocol for systematic reviews performed within this project has previously been published (Uter et al. 2021a), and registered under the PROSPERO registration number CRD42021238118 (Uter et al. 2021b). It is based on the Preferred Reporting Items for Systematic Reviews and Meta-Analysis Protocols (PRISMA-P) (Shamseer et al. 2015).

### Search strategy

A search was performed in Medline, Web of Science Core Collection, Cochrane Library, Toxicological Dossiers of the Scientific Committee on Consumer Safety (SCCS) of the European Commission, and of the German MAK Commission in the period from April to July 2021.

The search was composed of two modules: substance identifiers, and systemic/respiratory toxicity endpoints as shown in the supplemental Appendix A. Observational studies (case–control studies, prospective and retrospective cohort studies, cross-sectional studies, clinical series, case reports), and experimental studies with full text in English, published from 2000 and onwards were eligible for inclusion if providing information on the: 1) adverse respiratory effects of hair bleaches or PS in hairdressers and hairdressing apprentices, and 2) respiratory toxicity of PS in animal models.

**Fig. 1:**
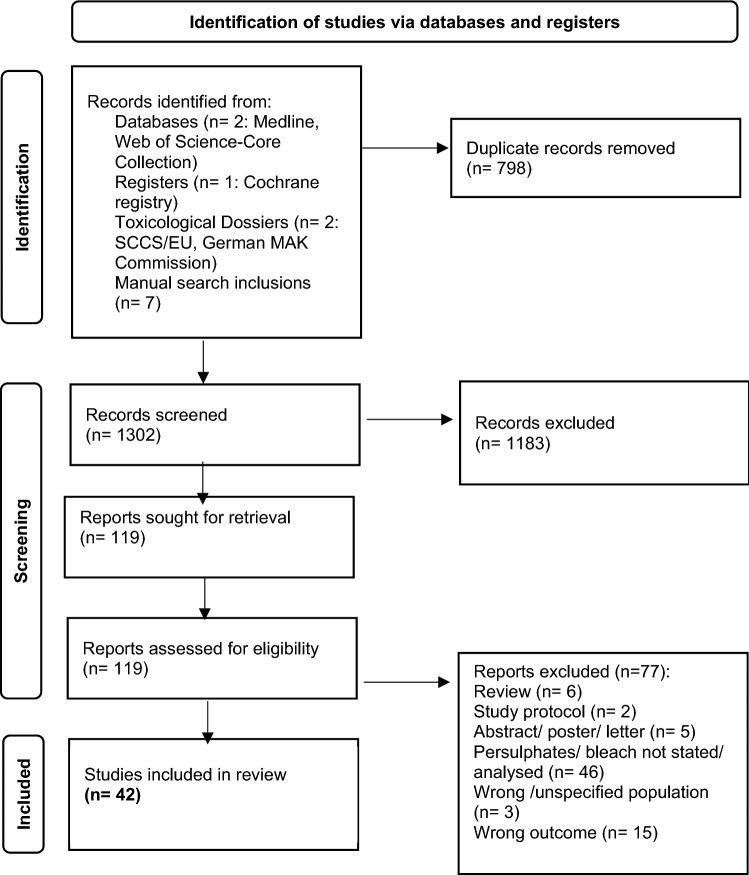
PRISMA flow-chart (Page et al. 2021)

The search results were imported from explored databases into Zotero libraries, where duplicates were removed before two independent reviewers assessed each study by title, key words and abstract using Rayyan (online tool: https:// rayyan. qcri. org/). Studies on which agreement for inclusion was reached, based on the predefined eligibility criteria, were retrieved for full-text analysis. Reasons for non-inclusion are summarised in the PRISMA flowchart (Fig. 1) (Page et al. 2021). The search was supplemented with hand searches of reference lists of already identified eligible studies, and a forward-snowballing citation analysis was conducted based on relevant sources found in the database searches.

### Data extraction

Two reviewers independently extracted the data from studies meeting the inclusion criteria using two separate publication record forms (PRFs) for observational and experimental studies. The following data were extracted for observational studies: Publication ID, year of study execution, country of origin, study design, methods, study setting and population involved, information on basic characteristics of participants (eg. age, gender), number of participants, number of positive outcome(s), and funding source. For experimental studies, publication ID, year of study execution, country of origin, study design, methods, study setting, test article, animals (species, sex, number), outcomes, and funding source were documented. Outcomes were extracted in subcategories: 1) respiratory symptoms, respiratory diseases, occupational respiratory diseases among hairdressers, and 2) respiratory outcomes in animal models exposed to PS. We were combining information in case of more than one publication reports on the same study reporting on different outcomes.

### Quality assessment

Criteria for the evaluation of quality and risk of bias for this systematic review were made by authors using four sources: i) mixed methods research appraisal (Pluye et al. 2009); ii) Cochrane collaboration (Sterne et al. 2020); iii) working group of the US EPA (Anon. 2003); and iv) animal studies guidance developed by the US National Toxicology Program’s Office of Health Assessment and Translation (OHAT) and outlined in OHAT’s risk of bias documentation (National Toxicology Program 2019). Criteria consisted of three parts regarding appropriate design, sampling and sample, justification of methodology (validity and standards), and justification/presentation of results. A maximal score of 15 was possible for clinical observational and experimental animal studies (case reports were not evaluated). Criteria were described in details in the supplemental Appendix B. A score yielding a proportion ≥ 70% (i.e. ≥ 10.5 points) was considered of good quality and a score < 70% to be of lower quality. Two reviewers who independently extracted the data from included studies also assessed the quality of studies, including the risk of bias.

### Data analysis

Owing to the heterogeneity of study designs, outcomes and methods, no attempt was made to quantitatively pool study results in terms of a meta-analysis. A narrative synthesis of data was done, focusing on prevalence, incidence, and risk estimates related to respiratory responses to PS exposure in hairdressers, as well as pathophysiological parameters documented in respiratory responses to PS in hairdressers and animal models. Summary tables present the main characteristics of the included studies, their findings as well as their quality ratings (Tables 1, 2, 3, 4 and 5).

**Table 1:** Reports of occupational asthma (OA) caused by persulphates (PS) in hairdressers from national surveys or registries (*n* = 4)

Author, year of publication, country	Study period, study design, respiratory outcome	Methods	*N* cases	Main outcomes	Quality assessment Total score*
Kopferschmitt-Kubler et al. (2002) France	1997 Cross-sectional OA 1-year report	Structured reports from physicians about OA	559 OA cases	Hairdressers 5.2% (4th place) PS as cause in OA in 4.1% of all cases (5th place)	**10.5**
Ameille et al. (2003) France	1996–1999 Retrospective cohort OA	OA cases reported to the national register	2178 OA cases	149 OA cases in hairdressers (4th place), PS as cause of OA in 137 cases; PS were cause of OA in 5.8% of all OA cases (5th place)	**13**
Orriols et al. (2006) Spain	2002 Cross-sectional Occupational respiratory diseases	Survey Reports about cases of occupational respiratory diseases from physicians	359 cases	Most common occupational respiratory disease was asthma in 174 cases (48.5%); PS as cause of OA in 21 (12.1%) cases (2nd place)	10
Moscato et al. (2014) Italy	Cross-sectional OA	Survey Reports about OA cases from allergologists	80 OA cases	15% of OA cases in hairdressers (2nd place) PS as cause in 11.1% of all cases (4th place)	8.5†

*Maximum score = 15; scores ≥ 70% of maximum score are in bold

†Studies with the indicated risk of bias

**Table 2:** Epidemiological studies confirming persulphates as cause of occupational asthma and/or occupational rhinitis in hairdressers, based on specific inhalation challenge as „gold “ diagnostic standard (*n* = 11 studies, *n* = 13 publications)

Author, year of publication, country	Study period, study design, respiratory outcome	Methods	*N* cases	*N* controls	Main outcomes	Quality assessment Total score*
Munoz et al. (2003) Spain	1997–2001 Prospective study OA	SPT to common inhalatory allergens, APS, PPS; total IgE; NSIC; SIC with PPS; PEF-monitoring; spirometry at follow-up	8 cases 5 hairdressers		Time between start of exposure and diagnosis: 15 y SIC + in 7; total IgE + in 6; pre-rhinitis in 6; SPT + to PS in 5 with increased total IgE IgE dependent mechanism of OA caused by PS suspected; diagnosis must be based on SIC	**14**
Munoz et al. (2004) Spain	1997–2002 Case–control OA	SPT to common inhalatory allergens, APS, PPS; total IgE; NSIC; SIC with PPS	Suspected OA 8 cases 5 hairdressers	8 other asthma cases 10 healthy subjects	Suspected OA: SPT + to PS in 4/8; SIC + in 6/8 (1 early, 6 late, 1 dual bronchial response) Other asthma cases: SIC + in 1/8 (late response)	**13.5**
Di Stefano et al. (2004) Italy, UK, USA	1993–2001 Retrospective cohort OA due to low molecular weight agents	spirometry, NSIC, PEF monitoring, SIC partially, SPT to common inhalatory allergens	98 OA cases (hairdressers not separated)		PS were cause of OA in 3 (3%) cases, with hairdressers as common occupation	9.5
Moscato et al. (2005) Italy	1996–2004 Retrospective cohort OA	SPT to common inhalatory allergens, latex, APS; patch test to hairdressing chemicals; total and specific IgE to common inhalatory allergens and latex), spirometry, NSIC, SIC with APS, sputum induction	47 hairdressers		OA established in 24 (51.1%) cases, PS as cause in 21 (87.5%); SPT to APS negative; Bronchial response in SIC + : early 4/late 14/dual 3; Eosinophylic airway inflammation prevailed in induced sputum	**12.5**
Airaksinen et al. (2008) Finland	1997–2003 Retrospective cohort ORh	SPT to common and occupational inhalatory allergens; SIC with hair bleach powder	2067 (hairdressers not separated)		SIC with bleach (*n* = 82): nasal response + in 8, bronchial response + in 11 cases; SPT + to PS 1/67; PS was most common agent causing ORh in hairdressers	10
Diab et al. (2009) Jonsson et al. (2009) Karedal et al. (2010) Sweden	Case–control ORh	SPT to common inhalatory allergens; SIC with PPS; nasal lavage, specific IgE to PS, blood tests, flow-citometry, PCR for gene IL-5, IL-13, IFN-Y	15 hairdressers with ORh to bleach	14 hairdressers without rhinitis 12 atopics (non-hairdressers)	Hairdressers with ORh had post challenge increase in nasal symptoms and amount of albumin in lavage; no positive SPT or specific IgE to PS Hairdressers with ORh and atopics differ in post SIC parameters in nasal lavage: increase in IL-13 only in atopics, in IFN-Y only in hairdressers with ORh, in IL-5 both; apolipoprotein A1 increased only in hairdressers with ORh	**12.5** **12.5** **14**
Moscato et al. (2010) Italy	1996–2008 Retrospective cohort OA, ORh	SPT to common inhalatory allergens, latex, APS; patch test to occupational allergens; NSIC; SIC with APS; nasal lavage, induced sputum	25 hairdressers with OA to PS		OA only established in 46%, OA + ORh in 53% SPT to APS negative; patch test to APS + in 8; Bronchial response in SIC: early 33%, late 66%; Nasal response in SIC: early 64%, late 36%; Eosinophils in nasal lavage and sputum in 90% of cases	**14**
Kronholm Diab et al. (2014) Sweden	Prospective study ORh	Clinical inteview, SPT to PPS, nasal lavage (ECP, albumin, triptase); diary; QoL questionnaire; SIC	17 hairdressers with ORh to bleach	19 hairdressers without rhinitis 10 atopics (non-hairdressers)	Hairdressers with ORh had increase in nasal symptom score and ECP in nasal lavage during 4 weeks at work, no change in SIC; SPT to PPS negative	**11.5**
Hagemeyer et al. (2015) Germany	2003–2014 Retrospective cohort OA	SPT to common inhalatory allergens, APS; NSIC, SIC with APS; patch test with APS; total IgE, eNO, thorax X-ray	8 OA cases caused by PS 7 hairdressers		Comparison of 2 SIC protocols (4 and 6 steps); 6 atopics; 4 SIC + , all late response, 1 SPT + to APS; 3 out of 4 patch test + to APS; eosinophils increased in blood in 8;	**12.5**
Foss-Skiftesvik et al. (2016) Denmark	2014–2016 Case–control OA, ORh	SPT to common inhalatory allergens, latex, chlorhexidin, APS, PPS, SPS; total IgE; NSIC, FeNO, SIC with PPS	20 hairdressers with nasal and bronchial symptoms	14 non-hairdressers with nasal and bronchial symptoms 40 healthy subjects	SIC + in 6/19 hairdressers with: normal total IgE, atopy 4/6, rhinitic response 6/6, bronchial response 2/6, immediate reaction 4/6, immediate + late 1/6, late 1/6, SPT negative to all three PS	**14.5**
Nielssen et al. (2016) Sweden	Cross-sectional ORh, OA	SPT to common inhalatory allergens and PPS, spirometry, SIC in chamber with 3 consecutive bleaching procedures (symptom score, peak nasal inspiratory flow, nasal lavage, blood measurements- haemoglobine, differential blood count, IL-6, IL-8, TNFα)	12 hairdressers with rhinitis to bleach (6 exposed to dust-free bleaching powder, 6 to regular bleaching powder)		Both groups develop asthma-like symptoms after exposure; no changes found in eye and nasal symptoms, spirometry, nasal flow; increase in neutrophils, lymphocytes, monocytes after SIC in both groups; IL-8 increase in nasal lavage in both groups	**10.5**

*PS* persulphates, *APS* ammonium persulphate, *PPS* potassium persulphate, *SPS* sodium persulphate, *OA* occupational asthma, *ORh* occupational rhinitis, *SPT* skin prick test, *SIC* specific inhalation challenge, *NSIC* nonspecific inhalation challenge, *FeNO* fraction of exhaled nitric oxides, *eNO* exhaled nitric oxides, *IgE* immunoglobulin E, *PEF* peak expiratory flow, *IL* interleukine, *IFN* *Y* interferon gamma, *TNF* α tumor necrosis factor alfa, *ECP* eosinophil cationic protein, *QoL* quality of life, *PCR* polymerase chain reaction

*Maximum score = 15; scores ≥ 70% of maximum score are in bold

**Table 3:** Case reports supportive for diagnosis of asthma, rhinitis, and anaphylaxis caused by persuphates in hairdressers (*n* = 8)

Author, year of publication, country	Age (years), females	Case history	Methods	Main outcome
Harth et al. (2006) Germany	26	Development of asthmatic symptoms after 8 working-years	SPT + to house dust mite, pollens, negative to APS; NSIC + ; SIC to APS + (only late reaction); patch test + to Ni, Co, PPD, APS, glycerol monothioglycolate	Occupational asthma case, relevance of contact sensitization for diagnosing airway disease was discussed
Figueiredo et al. (2008) Brazil	37	Development of nasal symptoms at work after 6 working-month, asthmatic symptoms after 3 working-years	SPT negative, including APS and PPS; total IgE normal; patch test + to colophonium, thimerosal, Ni; NSIC + ; SIC with PPS +	Occupational asthma due to PS can be diagnosed only by SIC
Bregnhoj and Søsted (2009) Denmark	29	Hairdresser with eczema and asthma developed 2 and 3 years after beginning to work, respectively	Patch test + to PPD, APS; SPT + to cat, horse, APS; neg.SPT to PPS	Eczema and asthma with type-1 and type-4 allergy caused by APS, without cross-reactivity to PPS in type-1 allergy
Pala et al. (2011) Italy	25	Onset of rhinitic symptoms and cough 2 years after beginning of work with bleach	SPT neg. to APS, NSIC negative, patch test + to Ni; total IgE normal; SIC + nasal response only, after SIC FeNO increase, eosinophil increase in nasal secretion and blood	Case of occupational rhinitis, suspected nonasthmatic eosinophylic bronchitis
Hoekstra et al. (2012) Nederlands	36	Rhinitis, asthma, and contact urticaria provoked by bleach at workplace	SPT + to APS and PPS; patch test + to APS and PPS after 20 min, specific IgE negative to APS	Case of systemic reaction to PS after skin contact; careful protocol should be employed- patch test read after 20 min, titration SPT; mechanism of immediate reaction unclear
Hougaard et al. (2012) Denmark	18 (apprentice)	Hand eczema and asthma developed 15 months after beginning education	SPT + to APS and PPS, positive PEF- monitoring, patch test + to APS	Occupational asthma and dermatitis caused by PS
Herin et al. (2012) France	38	Chest thightness, dyspnoea, noisy breathing, dysphonia, cough in association with bleaching procedures after 17 working-years	Total IgE normal, SPT negative to PS, NSIC negative, spirometry normal, SIC to PS negative, but symptoms like dysphonia, cough, sore throat occured; clinically vocal cord oedema was observed	Case of irritant vocal cord dysfunction after exposure to PS, occupational asthma excluded by SIC
Kleniewska et al. (2016) Poland	50	Rhinitis and contact urticaria at work after 15 working-years, attack of anaphylaxis (facial oedema, severe dyspnea) to PS in dental cement during dental procedure	Total IgE elevated, specific IgE + only to latex, negative to APS; NSIC negative; SPT + to APS, PPS, latex, grass; patch test + to APS; SIC to bleach product + after 15 min (sneezing and urticaria)	Case of allergic contact dermatitis, contact urticaria, and anaphylaxis to PS

*PS* persulphates, *APS* ammonium persulphate, *PPS* potassium persulphate, *SPT* skin prick test, *SIC* specific inhalation challenge, *NSIC* nonspecific inhalation challenge, *FeNO* fraction of exhaled nitric oxides, *IgE* immunoglobulin E, *PEF* peak expiratory flow, *Ni* nickel, *Co* cobalt, *PPD* p-phenylendiamine

*Maximum score = 15; scores ≥ 70% of maximum score are in bold

**Table 4:** Epidemiological studies assessing relation between respiratory symptoms/diseases are persulphate/bleach exposure (*n* = 9 studies, *n* = 10 publications)

Author, year of publication, country	Study period, study design, respiratory outcome	Methods	*N* hairdressers	*N* controls	Main outcomes	Quality assessment Total score*
Hollund et al. (2001), (2003) Norway	1995–1999 Prospective study Respiratory symptoms, atopy	Questionnaire, interview (for exposure assessment), total/specific IgE	91	80 office workers	Hairdressers over 40 years had significantly more symptoms than controls in a model adjusted for atopy and smoking: •Wheezing- 56 vs 24%, respectively, OR 3.3 (95% CI 1.0 to 11) •Breathlessness- 68 vs 33%, respectively, OR 3.9 (95% CI 1.1 to 14) Hairdressers had significantly more symptoms of wheezing, breathlessness, or runny nose from exposure to bleaching powder than controls in a model adjusted for atopy, age, and smoking: 44 vs 3%, respectively, OR 20 (95% CI 4.3 to 96) Hairdressers reported on average 13 chemically treated clients per week In 1999, former hairdressers reported significantly more respiratory symptoms when exposed to bleaching powder, compared with current hairdressers	**12.5** **12.5**
Albin et al. (2002) Sweden	1996–1997 Cross-sectional Asthma	Questionnaire	3957	4905 general population	Moderate effects on risk of asthma were found from hairdressing work (among never-smokers in comparison to controls in a model adjusted for calendar year, hay fever and region of residence: •Asthma incidence 4.4. vs 2.5 per 1000 person-years, respectively, IRR 1.6 (95% CI 1.1 to 2.2) The hairdressers most often performing hair bleaching treatments or using hair spray had, compared with the most infrequent users, a slightly, but not significantly higher incidence of asthma in a model adjusted for calendar year, hay fever, smoking and region of residence: •Bleaching- IRR 1.5 (95% CI 0.7 to 3.0) •Hair spraying- IRR 1.4 (95% CI 0.8 to 2.4) Two or more bleaching procedures per week reported 69% of hairdressers, 8 or more procedures 10%	**13**
Iwatsubo et al. (2003) France	1994–1997 Prospective study Respiratory symptoms, lung function	Questionnaire, spirometry, NSIC, expert workplace description	297 apprentices	248 office apprentices	In the initial phase, respiratory symptoms were significantly less frequent and lung function was better among hairdressing apprentices In the final phase, there was the same result for respiratory symptoms, but significant deterioration of lung function was found in hairdressing apprentices compared to controls. There was no significant correlation between change in lung function and specific hairdressing activities, including frequency of bleaching One or more bleaching procedure per day reported 41% of apprentices, 5 or more procedures 8%	**14**
Hashemi et al. (2010) Iran	Cross-sectional Respiratory symptoms, lung function	Questionnaire, spirometry	50	50 office workers	All respiratory symptoms (cough, breathless, wheezing, and phlegm) were significantly more prevalent in the hairdressers than in the control group (*P* < .001). Hairdressers reported that bleaching powder and hair spray were the most irritant chemicals that provoke respiratory symptoms. The impaired lung function tests in hairdressers followed symptom data	9.5
Lysdal et al. (2014) Denmark	1985–2007 Prospective cohort Respiratory symptoms	Questionnaire	5324 (all hairdressing graduates in study period)		Shortness of breath due to bleaching was reported in 27.1%; more ex-hairdressers ever had respiratory reaction to bleaching ( 30.2%) than current hairdressers (17.6%, OR 2.02, 95% CI 1.77–2.31). Respiratory reaction to bleaching was found significantly more in hairdressers with adulthood onset asthma (57.5%) than in hairdressers with childhood asthma (38.1%), and without asthma (24.4%)	**13.5**
Hassan and Bayomy (2015) Egypt	Cross-sectional Respiratory symptoms	Questionnaire	80	50 office workers	Hairdressers were more likely to report wheezes, chest tightness and cough than office workers (23.8 vs 8.0, 21.3 vs 8.0, and 25.0 vs 10%, respectively; *p* = 0.02, 0.04, and 0.03, respectively). Hairdressers who were more likely to report symptoms than controls were older, with higher body mass index and longer duration of work There were no significant associations between frequent bleaching and respiratory symptoms. One or more bleaching procedures per day reported 64% of hairdressers, 5 or more procedures 29%	10
Nemer et al. (2015) Palestine	2008–2013 Prospective study Respiratory symptoms, lung function	Questionnaire, spirometry, NH_3_ measurement	170 (initial phase) 161 (follow-up)		Current hairdressers developed more respiratory symptoms and larger lung function decline than former hairdressers during follow-up Hairdressers who applied bleaching more than 5 times per week showed a non-significant stronger decline of FEV_1_ compared with those who applied it less than 5 times per week. Five or more bleaching procedures per week reported 38% of hairdressers	**15**
Norlien et al. (2017) USA	2012 Cross-sectional Respiratory symptoms	Questionnaire	2058		Respiratory symptoms were reported by 46%. Relationship between asthma diagnosis and exposure results was not found. Use of bleach was reported by 87.3%	7.5†
Foss-Skiftesvik et al. (2017) Denmark	Cross-sectional Rhinitis, asthma	Questionnaire	504 apprentices	1400 general population	The 1-year prevalence of rhinitis symptoms was higher in hairdressing apprentices than in controls: •58.1% vs 46.6%, respectively, crude OR 1.59 (95%CI 1.30–1.98) Asthma symptoms were equally common. These findings were confirmed in models adjusted for smoking, education level, and degree of rurality Bleaching products were the most frequently reported cause of rhinitis and asthma symptoms in hairdressing apprentices	**13.5**

*OR* odds ratio, *IRR* incidence risk ratio, *CI* confidence internal, *FEV*_*1*_ forced expiratory volume in the first second, *IgE* immunoglobulin E, *NSIC* nonspecific inhalation challenge

*Maximum score = 15; scores ≥ 70% of maximum score are in bold

†Studies with the indicated risk of bias

**Table 5:** Experimental studies assessing response of the respiratory system to ammonium persulphate (*n* = 7)

Author, year of publication, country	Endpoints	Species, strain	Methods, study design	Main outcomes	Quality assessment Total score*
Signorin et al. (2001) USA	Subchronic inhalation toxicity	Rat, SD	13-wk inhalation OECD 413 (1981), Whole body exposure, recovery of 6 and 13 weeks	NOAEC 10.3 mg/m^3^; resp. irritation and ↑ lung weight at 25 mg/m^3^; subacute bronchial inflammation, mucus secretion and alveolar accumulation, regenerative hyperplasia of the bronchial and tracheal epithelium reversible 6 weeks post exposure	**14.5**
Dellabianca et al. (2010) Italy	NANC tracheal relaxation to EFS; cholinergic nerve-mediated contraction/ muscular response to exogenous carbachol or histamine	Guinea pig epithelium-free, isolated trachea	NANC relaxations to EFS at 3 Hz, involvement of inhibitory neurotransmitters, carbachol and histamine cumulative concentration–response curves Inhalation of AP aerosol (10 mg/m^3^ for 30 min/5 days/3 weeks)	Impaired nervous NANC inhibitory control in the guinea pig airways caused by AP inhalation. Marked inflammatory infiltration in the mucosa of tracheal segments	**13†**
De Vooght et al. (2010) Belgium and Spain	LPT in vitro, AHR to metacholine in the in vivo mouse model of chemical-induced asthma; BAL pulmonary inflammation markers; total serum IgE	Mouse, BALB/c	LPT with 1 and 5% AP or DMSO; Metacholine provocation test (whole body pletizmography *vs*. forced oscillation technique); BAL total and differential cell count, IFN-ƴ and IL-2, IL-4, IL-10, IL-13 in lymphocyte cultures from auricular, cervical and mediastinal lymph nodes; total serum IgE	LPT- lymphocytes of mice treated with AP showed two or three fold ↑ of incorporation of [^3^H]TdR upon incubation with AP; AHR—in AP sensitized and challenged mice: early ventilatory response immediately after intranasal challenge, ↑ bronchial reactivity: ↑ NPs in BAL ↑ serum IgE	**12†**
Olle-Monge et al. (2014) Spain	AHR to metacholine in the in vivo mouse model of chemical-induced asthma; BAL pulmonary inflammation markers; total serum Ig (IgE, IgG1 and IgG2a); lung histopathology	Mouse, BALB/c	Metacholine provocation test; BAL inflammation markers (IFN-ƴ and interleukins-2 (IL-2, IL-4, IL-5, IL-10, IL-13,IL-17A);Th2 related cytokines in homogenized lung tissue; total serum Ig mouse ELISA kit; BAL: differential cell counts; histological analysis of lung slides	Sustained increase of AHR to methacholine starting 1 h up to 4 days after AP challenge BAL: ↑ % of NPs 8 h after challenge, ↑ IL-10, IL-2 and IL-13 4 days after AP challenge, ↑ total serum IgE 4 days after challenge. Moderate inflammatory cell infiltration and alveolar macrophages in the lungs 8 h after challenge; at 4 days moderate peribronchiolar epitheilium hyperplasia; no collagen deposition	**13†**
Cruz et al. (2016) Spain	AHR to metacholine in the in vivo mouse model of chemical-induced asthma; BAL pulmonary inflammation markers; total serum IgE, IgG1 and IgG2a; lung pathology	Mouse, BALB/c	Metacholine provocation test; BAL: total and differential cell counts; total serum Ig mouse ELISA kit; histological analysis of lung slides	↑ AHR to methacholine, ↑ pulmonary inflammation 40 days after initial AP sensitization. BAL: ↑ % of NPs (returned to baseline 60 days after challenge); total serum IgE: ↑ on day 22 after dermal sensitization; total serum IgG1 and IgG2a ↑ from 45 days after dermal sensitization and remained high at 90 days; lungs: ↑ inflammatory cell infiltration and alveolar macrophages 60 days after sensitization, no collagen deposition	**12†**
Olle-Monge et al. (2017) Spain	AHR to metacholine in the in vivo mouse model of chemical-induced asthma; BAL pulmonary inflammation markers; total serum IgE; lung histopathology	Mouse, BALB/c	Metacholine provocation test; BAL: inflammation markers, total serum IgE; IFN-ƴ and interleukins-2 (IL-2, IL-4, IL-5, IL-10, IL-13 and IL-17A); histological analysis of lung slides, i.p. application of 200 µg of anti-IgE 1–5 mAb antibodies before intranasal AP challenge	Anti-IgE mAb treatment neutralized free serum IgE and abolished AHR 24 and 48 h after last challenge, BAL: ↓ total number of Eo, NPs and(IL)-13 after anti-IgE administration Lungs: anti-IgE-treated mice showed normal inflammatory patterns similar to control	**13†**
Dellabianca et al. (2020) Italy	Effect of PPAR-alfa receptor stimulation in preventing reduction in NANC tracheal caused by inhaled AP	Guinea pig epithelium-free, isolated trachea	NANC relaxations to EFS at 3 Hz in whole tracheal segments changes after AP inhalation (10 mg/m^3^) for 30 min for 5 days during 3 weeks, with and without PPAR-alfa agonist WY 14,643 or antagonist GW 6471 (0.36 µM/day p.o.)	PPAR-alfa agonist protects the NANC inhibitory system of the trachea from the effect of AP	**13.5†**

*NOAEC* no observed adverse effect concentration, *IgE* immunoglobulin E, *IgG* immunoglobulin G, *IL* interleukine, IFN Y interferon gamma,*Th2* T helper cells type 2, *mAb* monoclonal antibody, [3H]*TdR* radiolabeled thymidine, *NANC* non-adrenergic non-cholinergic, *EFS* electrical field stimulation, *PPAR* peroxisome proliferator-activated receptor, *LPT* lymphocyte proliferation test, *AHR* airway hyperresponsiveness, *BAL* bronchoalveolar lavage, *DMSO* dimehyl sulfoxide, *NPs* neutrophyls, *Eo* eosinophils, *ELISA* enzyme-linked immunoassay, *SD* sprague dawley, *BALB* bagg albino, *OECD* Organisation for Economic Cooperation and Development

*Maximum score = 15; scores ≥ 70% of maximum score are in bold

†Studies with the indicated risk of bias; AP- Ammonium persulphate

## Results

**Fig. 2:**
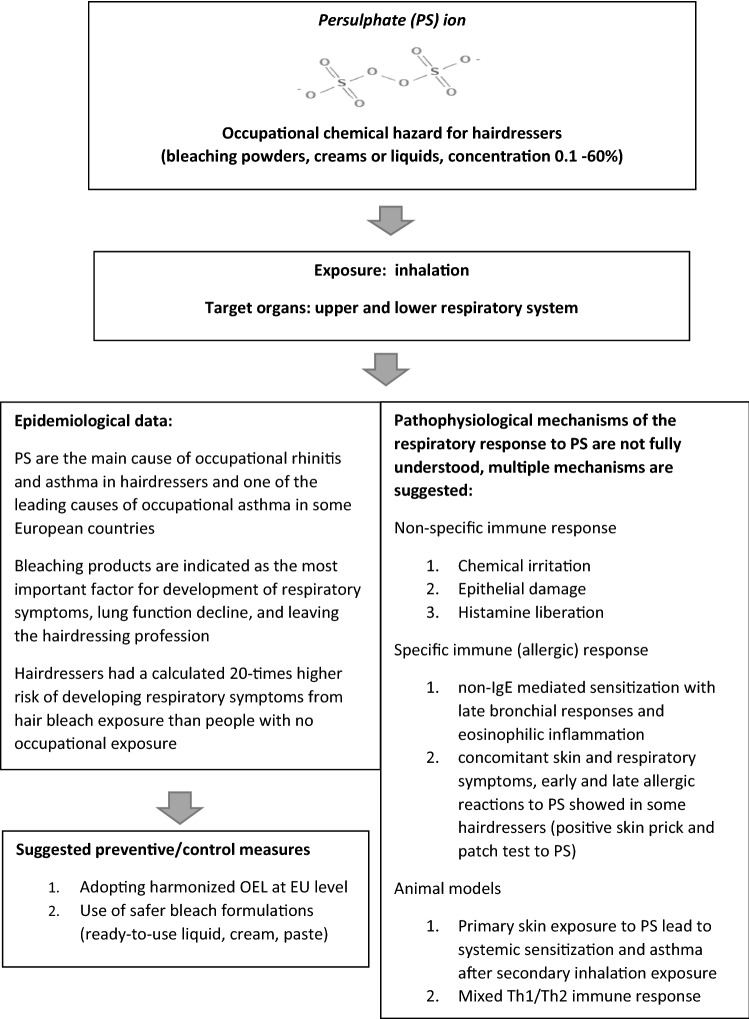
Main goals and outcomes of the systematic review

The process of identification, screening and inclusion of studies (PRISMA flowchart) is presented in Fig. 1. In total, 42 studies were included consisting of: (1) clinical observational studies and case reports (*n* = 19) describing occupational respiratory diseases caused by PS; (2) reports from national surveys or registries of occupational diseases (*n* = 4); (3) clinical observational studies assessing relation between respiratory symptoms/diseases and PS/bleach exposure (*n* = 10); and (4) experimental studies assessing response of the respiratory system to PS exposure in animal models (*n* = 7). A score ≥ 70% was found in 27 out of 34 (79.4%) studies assessed for quality (Tables 1,2,4 and 5). Observational studies of poor quality were included in the narrative synthesis of data if there was no indication of bias. Indication of selection bias was not found, while information bias was suspected in two studies based on web/e-mail questionnaires (Moscato et al. 2014; Norlien et al. 2017). The study of Norlien et al. (2017) was not taken into consideration, while the study of Moscato et al. (2014) was interpreted along with other studies based on national surveys and registries because its results were in line with other studies. Unclear risk of selection bias due to the poor reporting was identified in four experimental studies where randomization was not described (Cruz et al. 2016; Olle-Monge et al. 2014, 2017; De Vooght et al. 2010), and unclear risk of information bias was found in three studies without details on APS purity (Dellabianca et al. 2010, 2020; De Vooght et al. 2010). However, these are all mechanistic studies with robust methodology and consistent outcomes in line with the results of similar studies where risk of bias was not identified and are consequently included in this review. Main results are summarized in Fig. 2.

## Occupational rhinitis and asthma caused by persulphates

### National surveys and registries of occupational diseases

Four studies reporting data about occupational respiratory diseases from national registries and surveys were found (Kopferschmitt-Kubler et al. 2002; Ameille et al. 2003; Orriols et al. 2006; Moscato et al. 2014) (Table 1). The largest study described data from the French national registry including all occupational asthma (OA) cases (*n* = 2178) for the period 1996–1999. Hairdressers were shown as the fourth most frequent occupation with a total of 149 OA cases which were in 137 cases caused by PS. In a list of causative factors for OA, PS were listed as a fifth most frequent cause (5.8% of all OA cases) (Ameille et al. 2003). In a survey from Spain on occupational respiratory diseases (*n* = 359) reported in 2002, PS were shown as a cause of OA in 12.1% of OA cases, making this agent the second most frequent cause of OA. Occupation specific incidence rate for occupational asthma was 108 per million person-years in hairdressers in comparison to 4.9 in white-collar workers, and 96 in cleaners (Orriols et al. 2006). An Italian web-survey on OA (*n* = 80) showed hairdressers as the second most frequent occupation, and PS as the fourth most frequent causative agent for OA (cause of OA in 11.1% of all OA cases) (Moscato et al. 2014).

### Clinical observational studies

Included clinical observational studies confirming PS or hair bleaches as a cause of occupational respiratory diseases in hairdressers were based on specific inhalation challenge (SIC) as a diagnostic „gold standard”. Studies published in the period 2003–2005 considered only the development of OA and in studies from 2008 to 2016 occupational rhinitis (ORh) was also examined. Study design and main outcomes from 11 clinical observational studies are shown in Table 2.

Five studies were designed as retrospective cohorts of patients (Di Stefano et al. 2004; Moscato et al. 2005, 2010; Airaksinen et al. 2008; Hagemeyer et al. 2015). A study from Italy described the biggest retrospective cohort of hairdressers who underwent a diagnostic procedure for OA in the period 1996–2004 (*n* = 47), with OA diagnosed in 24 (51.1%) cases, and APS as a cause of OA in 21 out of 24 (87.5%) cases (Moscato et al. 2005). This cohort was supplemented in the second study for the period 1996–2008 with 26 hairdressers with established diagnosis of OA by means of a diagnostic protocol for ORh caused by APS, revealing 12 (46.2%) cases of OA, and 14 (53.8%) cases of OA and ORh. A retrospective cohort of patients with OA due to low molecular weight agents from Italy (*n* = 98) for the period 1993–2001 confirmed PS as cause of OA in 3 (3%) cases, with hairdressers as common occupation (Di Stefano et al. 2004). A large retrospective cohort of patients who underwent diagnostic procedure for ORh from Finland (*n* = 2067) reported PS as the most common agent causing rhinitis in hairdressers (Airaksinen et al. 2008). From 82 specific inhalation challenges with PS, 8 positive nasal responses, and 11 positive bronchial responses were observed.

Nine studies performed skin prick tests to APS and/or PPS as a part of the diagnostic protocol for OA and/or ORh (Table 2). Skin prick tests with PS were negative in all tested subjects in six studies (Moscato et al. 2005; Moscato et al. 2010; Diab et al. 2009; Kronholm Diab et al. 2014; Foss-Skiftesvik et al. 2016; Nielssen et al. 2016), and were positive in one out of eight, four out of eight, and five out of eight subjects in three studies (Munoz et al. 2003; Munoz et al. 2004; Hagemeyer et al. 2015). Specific IgE to PS was retrieved in only one study, with negative result (Diab et al. 2009). Patch tests with PS were performed in three studies with positive results in the minority of subjects (Moscato et al. 2005, 2010; Hagemeyer et al. 2015). In study of Moscato et al. (2010), 8 out of 26 cases were patch tested positive to APS with an additional diagnosis of occupational allergic contact dermatitis, and skin symptoms preceded respiratory symptoms in all cases.

The pattern of bronchial response in positive SIC with PS was described in 5 studies, and late responses prevailed in all studies making around two thirds of all responses (Moscato et al. 2005; Moscato et al. 2010; Munoz et al. 2004; Hagemeyer et al. 2015; Foss-Skiftesvik et al. 2016). Two studies described a pattern of nasal response in positive SIC, revealing a different outcome with domination of early response (Moscato et al. 2010; Foss-Skiftesvik et al. 2016). Pattern of eosinophilic inflammation was found during SIC in induced sputum and nasal lavage in three studies (Moscato et al. 2005, 2010; Kronholm Diab et al. 2014), and one study found increased eosinophils in blood (Hagemeyer et al. 2015).

A case–control study with atopic control subjects not occupationally exposed to hair bleach revealed that nasal SIC with PS affects hairdressers with rhinitis to bleach as well as atopics. However, nasal response showed some differences. Hairdressers with rhinitis to bleach showed increases in apolipoprotein A1, IL-5, IFN-Y in post SIC nasal lavage, while, in atopics, an increase in IL-5 and IL-13 was found (Diab et al. 2009; Jonsson et al. 2009; Karedal et al. 2010). One study showed positive SIC to PS in one out of eight control subjects with asthma not caused by PS, no positive SIC in ten healthy control subjects, in contrast to six (among these five hairdressers) out of eight subjects with asthma and occupational exposure to PS (Munoz et al. 2004). The study from Denmark included 19 hairdressers with work-related rhinitis and/or asthma, 12 symptomatic controls (10 with allergic asthma and rhinitis and 2 with non-allergic asthma), and 40 healthy controls. None of the symptomatic controls had a nasal or bronchial response to SIC with PPS. Six hairdressers had nasal and two bronchial responses. All three groups showed non-specific non-IgE-mediated histamine release to PS in histamine-release tests (Foss-Skiftesvik et al. 2016).

### Case reports

Literature search revealed eight case reports (Harth et al. 2006; Figueiredo et al. 2008; Bregnhøj and Søsted 2009; Pala et al. 2011; Hoekstra et al. 2012; Hougaard et al. 2012; Herin et al. 2012; Kleniewska et al. 2016) providing supporting evidence on OA and ORh in hairdressers caused by PS (Table 3). Two case reports described both contact dermatitis and asthma with type I and type IV allergic reactions to APS in terms of positive skin prick and patch tests (Hougaard et al. 2012; Bregnhøj and Søsted 2009). Another case report confirmed a diagnosis of irritant vocal cord dysfunction after exposure to PS (occupational asthma was excluded by SIC) (Herin et al. 2012). Two case reports presented development of systemic hypersensitivity reactions provoked by PS in hairdressers. In one case following rhinitis and asthma, contact urticaria after skin contact also developed (Hoekstra et al. 2012). In another case report, a hairdresser with allergic contact dermatitis and rhinitis to PS developed contact urticaria at work, and suffered from anaphylaxis (facial oedema, severe dyspnoea) after non-occupational contact with PS from dental cement (Kleniewska et al. 2016).

## Exposure to persulphate/bleach in hairdressers and respiratory symptoms/lung function decline

Literature search revealed nine epidemiological studies assessing exposure and development of respiratory symptoms or diseases, and changes in lung function in hairdressers in relation to hair bleach exposure (Table 4). All studies used a questionnaire as the methodological approach, and three studies added lung function measurement. However, quantitative analysis was not possible due to the differences in questionnaires regarding form and implementation (web, e-mail, printed, fill-out by participants or investigators), and the way the type, onset and duration of respiratory symptoms was documented.

Five cross-sectional studies compared hairdressers (Albin et al. 2002; Hashemi et al. 2010; Hassan and Bayomy 2015; Norlien et al. 2017) or hairdressing apprentices (Foss-Skiftesvik et al. 2017) to a control group of subjects not occupationally exposed to hairdressing chemicals. A study performed in Sweden examined asthma incidence in a large sample of hairdressers (*n* = 3957) and control subjects from the general population (*n* = 4905) in the period 1996–1997. A moderately increased risk for asthma was found in non-smoking hairdressers in comparison to controls in a model adjusted for the calendar year, hay fever and region of residence (incidence rate ratio [IRR] 1.6, 95% CI 1.1 to 2.2; asthma incidence 4.4 *vs* 2.5 per 1000 person-years, respectively). Additionally, a slightly, but not significantly, higher risk for asthma was found in hairdressers who most often used bleaches (IRR 1.5, 95% CI 0.7 to 3.0; asthma incidence 4.7 per 1000 person-years) and hair sprays (IRR 1.4, 95% CI 0.8 to 2.4; asthma incidence 4.7per 1000 person-years) (Albin et al. 2002). A study on hairdressing apprentices (*n* = 504) and a control group (*n* = 1400) from Denmark found a significantly higher 1-year prevalence of rhinitic symptoms in hairdressing apprentices than controls (58.1 vs 46.6%, respectively; crude OR 1.6, 95% CI 1.3–1.9), while such difference was not found for asthma. These findings were confirmed in models adjusted for smoking, education level, and degree of rurality. Bleaching products were the most frequently reported cause of respiratory symptoms by hairdressing apprentices (Foss-Skiftesvik et al. 2017). Studies from Egypt and Iran (Hashemi et al. 2010; Hassan and Bayomy 2015) found significantly more prevalent self-reported respiratory symptoms in hairdressers than in control subjects, but only in one study bleaching powder and hair sprays were reported as the most irritant chemicals provoking respiratory symptoms (Hashemi et al. 2010), while another study did not find such relation (Hassan and Bayomy 2015).

Four studies had a prospective design (Hollund et al. 2001, 2003; Iwatsubo et al. 2003; Lysdal et al. 2014; Nemer et al. 2015), and one of them was performed on hairdressing apprentices (Iwatsubo et al. 2003). A study from Norway with a follow-up from 1995 to 1999 revealed that hairdressers over 40 years of age reported significantly more symptoms of wheezing (56 vs 24%, respectively, OR 3.3; 95% CI 1.0 to 11) and breathlessness (68 vs 33%, respectively, OR 3.9; 95% CI 1.1 to 14) than controls (office workers) in a model adjusted for atopy and smoking. Hairdressers also reported significantly more symptoms of wheezing, breathlessness, or runny nose from exposure to bleaching powder than controls (44 vs 3%, respectively, OR 20; 95% CI 4.3 to 96) in a model adjusted for atopy, age, and smoking, limited to hairdressers and those office workers reporting use of any hair treatment products (Hollund et al. 2001). This study, and a study from Denmark (cohort of 5324 hairdressers followed in a period 1985–2007 by questionnaire) showed that exposure to bleaching products was the most important factor for development of respiratory symptoms and for leaving the hairdressing profession (Hollund et al. 2003; Lysdal et al. 2014). A study from Palestine with the follow-up of hairdressers in the period 2008–2013 (n = 170) showed that hairdressers who applied bleach more than five times per week had a slightly stronger, but statistically not significant decline of FEV_1_ (forced expiratory volume in the first second) compared to those who applied it less frequently (Nemer et al. 2015). A prospective study on hairdressing (n = 297) and office (n = 248) apprentices from France in the period 1994–1997 showed the deterioration of lung function in hairdressing apprentices during the follow-up period which was not found in office apprentices, but no significant correlation was found between change in lung function and specific hairdressing activities, including frequency of bleaching (Iwatsubo et al. 2003).

Three studies showed the self-reported frequency of performing bleaching procedure in hairdressers (Albin et al. 2002; Hassan and Bayomy 2015; Nemer et al. 2015), and one study in hairdressing apprentices (Iwatsubo et al. 2003). A study from Sweden showed that 69% of hairdressers performed bleaching procedure ≥ 2 times per week, and 10% ≥ 8 times per week (Albin et al. 2002). An Egyptian study found that 64% of hairdressers performed hair bleaching ≥ 1 time per day, and 29% ≥ 5 times per day (Hassan and Bayomy 2015). A Palestinian study showed that 38% of hairdressers reported bleaching procedure ≥ 5 times per week (Nemer et al. 2015). A French study found that 49% of hairdressing apprentices performed hair bleaching ≥ 1 time per day, and 8% ≥ 5 times per day (Iwatsubo et al. 2003). Additionally, a study from Norway reported hairdressers performing chemical treatment of hair (using hair dye, and bleaching powder altogether) in 13 clients per week on average (Hollund et al. 2001).

## Experimental studies in animal models evaluating respiratory response to persulphates

Seven experimental studies assessing inhalation toxicity and mechanisms of respiratory response to PS exposure in animal models were included (Table 5). This search identified only one 90-day inhalation toxicity study in rats exposed to APS conducted in line with Organisation for Economic Co-operation and Development Guidelines for testing of chemicals (OECD TG) 413 (Signorin et al. 2001). The no-observed-adverse-effect concentration (NOAEC) was 10.3 mg/m^3^, based on clinical signs, decreased body weights, elevated lung weights and microscopic lesions of the trachea and bronchi/ bronchiole, which were evident at the lowest-observed-adverse-effect concentration (LOAEC) of 25.0 mg/m^3^ (the highest dose tested).

Two studies showed that high concentrations of inhaled APS inhibit non-adrenergic, non-cholinergic (NANC) relaxation in guinea-pig isolated trachea suggesting the role of APS inhalation in airway tone regulation (Dellabianca et al. 2010, 2020).

The potential of APS of triggering an asthma-like response based on dermal sensitization and intranasal challenge was demonstrated in four in vivo studies, in the validated mouse model of chemical-induced non-atopic asthma (DeVooght et al. 2010; Cruz et al. 2016; Olle-Monge et al. 2014, 2017). Several features of human OA were induced one day after intranasal instillation of APS in dermally sensitized mice: airway hyperresponsiveness (AHR), neutrophilic inflammation, increased levels of total serum IgE, T and B cell proliferation and increased levels of Th2 cytokines (interleukin (IL)-4, IL-10 and IL-13) (DeVooght et al. 2010). A study investigating the time course of immunologic and respiratory responses after dermal sensitization showed that respiratory responsiveness to methacholine tends to persist even 60 days after initial APS sensitization, that is much longer than inflammation. There was evidence of systemic sensitization with an increase in IgE at early stages (15 days after initial dermal application), while high IgG levels appeared later (Cruz et al. 2016). Persistence of asthmatic response in APS-treated mice was confirmed in other two studies by Olle-Monge et al. (2014, 2017). AHR appeared immediately and a sustained increase lasted up to 4 days after the challenge. In BAL fluids, a significant increase in the percentage of neutrophils, but no eosinophils were found 8 h after the challenge, persisting for 24 h. Increased levels of IL-2, IL-10 and IL-13 in BAL fluid and IL-5 in tissue homogenate in AP-treated mice suggested a mixed Th1-Th2-type immune response in sensitized mice. Total serum IgE was slightly increased 4 days after the AP challenge, returning to baseline level 1 week later, while IgG levels gradually increased further for 4–15 days. Anti-IgE monoclonal antibody treatment almost completely neutralized free serum IgE, abolished AHR, significantly reduced the total number of eosinophils and neutrophils and IL-13 levels in the BAL 24 h and 48 h after the last challenge (Olle-Monge et al. 2017).

## Discussion and conclusions

Hairdressers are exposed to hair bleaches significantly more often than their clients or consumers using hair-bleaching products at home. About two-thirds of hairdressers and about half of hairdressing apprentices reported performing bleaching procedures two or more times per week, or one or more times per day (Albin et al. 2002; Hassan and Bayomy 2015; Iwatsubo et al. 2003). Clients usually do not have hair bleaching performed more than once in a month. Studies from Scandinavian countries showed an about 1.5 times higher risk for self-reported rhinitis symptoms in hairdressing apprentices, and for self-reported asthmatic symptoms in hairdressers, in comparison to controls occupationally not exposed to hair bleaches (Foss-Skiftesvik et al. 2017; Albin et al. 2002). The risk for asthma symptoms in hairdressers is increasing with age, being about 3.5 times higher in hairdressers aged 40 years or more than in matched controls (Hollund et al. 2001). Bleaching products were indicated as the most important factor for the development of respiratory symptoms, lung function decline, and leaving the hairdressing profession (Albin et al. 2002; Hashemi et al. 2010; Hollund et al. 2003; Lysdal et al. 2014; Nemer et al. 2015; Iwatsubo et al. 2003; Foss-Skiftesvik et al. 2017). A study from Norway showed that hairdressers had a 20 times higher risk to develop respiratory symptoms from exposure to bleaching powder than controls occupationally not exposed to hair bleaches (Hollund et al. 2001). PS are indicated as the main cause of ORh and OA in hairdressers (Moscato et al. 2005, 2010; Airaksinen et al. 2008; Ameille et al. 2003), and one of the leading causes of OA in some European countries, especially in France, Italy and Spain (Moscato et al. 2014; Ameille et al. 2003; Orriols et al. 2006). In this respect, this review builds on evidence from retrospective cohorts of patients undergoing a specific inhalatory challenge as a “gold standard” in confirming OA as well as from data from national registries for OA. A case report added a diagnosis of irritant vocal cord dysfunction after exposure to PS to the list of occupational respiratory diseases that may be provoked by PS in hairdressers (Herin et al. 2012). Two case reports presented development of systemic hypersensitivity reactions provoked by PS in terms of contact urticaria and anaphylaxis in hairdressers suffering from respiratory disorders caused by PS, suggesting that hairdressers with respiratory responses to PS should be closely monitored in case of persisting PS exposure at the workplace (Hoekstra et al. 2012; Kleniewska et al. 2016).

Pathophysiological mechanisms of the respiratory response to PS are not fully understood. Experimental studies of APS-induced respiratory hyperreactivity suggest different patterns of respiratory inflammation and hyperreactivity due to the chemical irritation, damage to the mucosal membranes and non-specific histamine liberation caused by PS. An irritative (non-specific) inflammatory pattern was supported by studies showing respiratory responses to inhalatory challenges with PS in a small proportion of symptomatic control subjects (atopics or asthmatics) not occupationally exposed to PS (Diab et al. 2009; Jonsson et al. 2009; Karedal et al. 2010; Munoz et al. 2004). However, inflammatory nasal responses differed qualitatively between exposed hairdressers and symptomatic controls (Diab et al. 2009; Jonsson et al. 2009; Karedal et al. 2010; Munoz et al. 2004), supporting the existence of additional pathophysiological patterns, like specific allergic mechanisms. Pathways of asthma-like responses based on primary dermal sensitization and later intranasal challenge with APS were investigated in the mouse model of chemical-induced non-atopic asthma (DeVooght et al. 2010; Cruz et al. 2016; Olle-Monge et al. 2014, 2017). APS caused an early respiratory response followed by prolonged airway hyperreactivity, suggesting that dermal contact with PS may lead to airway inflammation and asthmatic symptoms, and systemic sensitization with a short-term increase in IgE at early stages, and high IgG levels appearing later. A mixed Th1-/Th2-type immune response was suggested in sensitized mice with increased levels of IL-2, IL-10 and IL-13 in bronchoalveolar lavage fluid. However, the majority of retrieved epidemiological studies do not support Type I or IgE-mediated allergic reaction as a pattern of respiratory response to PS, reporting negative skin prick tests with PS, and negative specific IgE to PS (Moscato et al. 2005; Moscato et al. 2010; Diab et al. 2009; Kronholm Diab et al. 2014; Foss-Skiftesvik et al. 2016; Nielssen et al. 2016). The pattern of bronchial response to PS was mostly in favour of late responses with eosinophilic inflammation supporting development of specific non IgE-mediated sensitization in some exposed individuals (Moscato et al. 2005; Moscato et al. 2010; Munoz et al. 2004; Hagemeyer et al. 2015; Kronholm Diab et al. 2014; Foss-Skiftesvik et al. 2016). Due to the unclear, and possibly several co-existing pathophysiological pathways of respiratory responses to PS, the specific inhalation challenge is clearly indicated as the only diagnostic test which can establish a diagnosis of ORh and OA caused by PS (Munoz et al. 2003; Figueiredo et al. 2008).

A recent systematic review on animal models supported experimental evidence that skin exposure to low molecular weight agents such as PS may lead to systemic sensitization and subsequent development of asthma following inhalation exposure (Tsui et al. 2020). Scarce epidemiological and clinical evidence also supports a co-existence of skin and respiratory responses to PS, with dermatitis mostly found prior to the development of asthma, as well as concomitant Type I and Type IV allergic reactions to APS (positive skin prick and patch tests) (Moscato et al. 2010; Hougaard et al. 2012; Bregnhøj and Søsted 2009). An important role for skin barrier and skin exposure in the development of Th2-immune response and the subsequent development of respiratory disorders was suggested, but is not sufficiently defined in humans (Redlich and Herrick 2008; Cruz et al. 2016). So far, a clear distinction persists between skin and respiratory sensitizers and their clinical outcomes (contact dermatitis and rhinitis/asthma, respectively), including separate diagnostic algorithms.

While skin contact with PS can be significantly reduced by protective gloves, inhalatory exposure is a greater challenge regarding personal protective equipment or other safety at work measures. Studies suggest no increased risk of occupational respiratory disease from workplace exposures of up to 1 mg/m^3^ of APS (Signorin et al. 2001; Merget et al. 1996). The American Conference for Governmental Industrial Hygienists defined the 8-h time-weighted average OEL for PS at 0.1 mg/m^3^ (American Conference for Governmental Industrial Hygienists 1998), which is also accepted in several EU countries (ANSES 2019), but not set by regulatory authorities at EU level. Exposure to PS could fluctuate through time due to the changes in life-styles and fashion, and could differ between countries due to the dominant hair colour in the population and/or fashion habits. Although not yet confirmed in actual exposure studies, it is expected that substituting conventional bleach powder with cream, paste, fat droplet-adsorbed (so-called “dust-free”) powder, or ready-to-use liquid formulations will reduce PS exposure when mixing bleaching ingredients. However, as shown by Nielssen et al. (2016), emissions of PS also occur during application and probably during removal of bleach. Unfortunately, literature data are insufficient in this respect, as well as regarding prospective studies on the prevalence and incidence of occupational PS rhinitis and asthma in hairdressers through the last 20 years.

Strengths of this review are the systematic methods including a priori registered and published protocol (Uter et al. 2021a,b), good quality of the majority of included studies, and a qualitative synthesis of data from a broad methodological scope, i.e. clinical, epidemiological and experimental studies.

The limitation of this study is in heterogeneity of included studies by methods and outcomes which did not enable quantitative data synthesis (meta-analysis). A certain publication bias was noted in terms of a lack of epidemiological data from the last 10 years. This can compromise the relevance of included studies on current occupational conditions regarding hairdressers’ procedures and used products.

To conclude, hairdressers are occupationally more exposed to PS than the general population using hair bleach, with a calculated 20 times higher risk of developing respiratory symptoms from PS exposure than people with no occupational exposure. PS are the main cause of occupational rhinitis and asthma in hairdressers and one of the leading causes of occupational asthma in some European countries. Preventive safety at work measures for reducing inhalatory exposure to PS in hair salons should be re-evaluated and implemented, including adopting a harmonized OEL at EU level. Use of safer bleach formulations and further research in this field should be encouraged. For the last 20 years, epidemiological data regarding adverse respiratory effects of PS in hairdressers are generally scarce, and the lack of well-conducted cohort studies at EU level is particularly evident.

## Supplementary Information

Below is the link to the electronic supplementary material.

Supplementary file1 (DOCX 15 KB)

Supplementary file2 (DOCX 21 KB)
