# Supplementary material for: Respiratory toxicity of persulphate salts and their adverse effects on airways in hairdressers: a systematic review
Source: Int Arch Occup Environ Health. 2022 Mar 22;95(8):1679–702. doi: 10.1007/s00420-022-01852-w (PMC9489562; doi:10.1007/s00420-022-01852-w)
Supplement: Supplementary file 1 — Supplementary file1 (DOCX 15 KB) [file 420_2022_1852_MOESM1_ESM.docx]

**Appendix A**

**Substance identifiers:**

## Ammonium persulfate (INCI)

Shorthand: **APS**.

- Ammonium persulfate
- 7727-54-0
- Peroxydisulfuric acid ([(HO)S(O)_2]_2O_2), diammonium salt (8CI,9CI)
- Ammonium peroxidodisulfate
- Ammonium peroxydisulfate
- Ammonium peroxydisulfate ((NH_4)_2S_2O_8)
- Ammonium peroxysulfate
- Bis(ammonium) peroxodisulfate
- Diammonium peroxydisulfate
- Diammonium peroxydisulphate
- Diammonium persulfate

## Potassium persulfate (INCI)

Shorthand: **PPS**.

- Potassium persulfate
- 7727-21-1
- Peroxydisulfuric acid ([(HO)S(O)_2]_2O_2), dipotassium salt (9CI)
- Dipotassium peroxodisulfate
- Dipotassium peroxydisulfate
- Dipotassium persulfate
- Potassium dipersulfate
- Potassium peroxydisulfate
- Potassium peroxydisulfate (K_2(S_2O_8))
- Potassium peroxydisulphate

## Sodium persulfate (INCI)

Shorthand: **SPS**.

- Sodium persulfate
- 7775-27-1
- Peroxydisulfuric acid ([(HO)S(O)_2]_2O_2), disodium salt (8CI,9CI)
- Sodium peroxydisulfate (6CI)
- Disodium peroxodisulfate
- Disodium peroxydisulfate
- Disodium persulfate
- Sodium dipersulfate
- Sodium peroxodisulfate
- Sodium peroxydisulfate (Na_2S_2O_8)
- Sodium persulfate (Na_2S_2O_8)

**Respiratory/systemic toxicity endpoints:**

Endpoints include the relevant MeSH terms and common medical language synonyms listed below:

Allergens[MeSH] OR Irritants[MeSH] OR allergic OR irritative OR Respiration Disorders[MeSH] OR respiratory OR Inhalation[MeSH] OR Rhinitis[MeSH] OR Asthma OR Neoplasms[MeSH] OR cancer OR Carcinogens[MeSH] OR Biomarkers, Tumor[MeSH] OR Carcinogenicity Tests[MeSH] OR Mutagens[MeSH] OR Mutagenicity Tests[MeSH] OR genotoxicity OR Reproductive Health[MeSH] OR reproductive toxicity OR reprotoxic OR Pregnancy Outcomes[MeSH] OR Pregnancy Complications[MeSH] OR Pregnancy[MeSH] OR Infertility[MeSH] OR Congenital Abnormalities[MeSH] OR birth defect OR congenital malformations OR Abortion, Spontaneous[MeSH] OR Developmental Disabilities[MeSH] OR developmental toxicity OR Menstruation Disturbances[MeSH] OR Spermatogenesis[MeSH] OR Fertility[MESH] OR Fecundability OR Time to pregnancy OR low birth weight OR Endocrine Disruptors[MeSH] OR Endocrine System Diseases[MeSH] OR Toxicity Tests[MeSH] OR Toxicity Tests, Acute[MeSH] OR Toxicity Tests, Subacute[MeSH] OR Toxicity Tests, Chronic[MeSH] OR Toxicity Tests, Subchronic OR dermal absorption OR Occupational Diseases[MeSH] OR work related OR hairdresser* OR hairdressing
