# Supplementary material for: Respiratory toxicity of persulphate salts and their adverse effects on airways in hairdressers: a systematic review
Source: Int Arch Occup Environ Health. 2022 Mar 22;95(8):1679–702. doi: 10.1007/s00420-022-01852-w (PMC9489562; doi:10.1007/s00420-022-01852-w)
Supplement: Supplementary file 2 — Supplementary file2 (DOCX 21 KB) [file 420_2022_1852_MOESM2_ESM.docx]

**Appendix B.**

**Criteria for the evaluation of quality and risk of bias for the systematic reviews addressing health effects of hair cosmetic ingredients**

# **References**

Anon (2003) A Summary of General Assessment Factors for Evaluating the Quality of Scientific and Technical Information - Prepared for the U.S. Environmental Protection Agency by members of the Assessment Factors Workgroup, a group of the EPA’s Science Policy Council. Washington, DC: US EPA.

National Toxicology Program (2019) Handbook for conducting a literature-based health assessment using office of health assessment and translation (OHAT) approach for systematic review and evidence integration. Available at: <https://ntp.niehs.nih.gov/ntp/ohat/pubs/handbookmarch2019_508.pdf>. Accessed 22.12.2021.

Pluye P, Gagnon M-P, Griffiths F, Johnson-Lafleur J (2009) A scoring system for appraising mixed methods research, and concomitantly appraising qualitative, quantitative and mixed methods primary studies in Mixed Studies Reviews. Int J Nurs Stud 46(4):529–46. doi: 10.1016/j.ijnurstu.2009.01.009

Sterne JAC, Hernán MA, McAlleenan A, Reeves BC, Higgins JPT (2020) Chapter 25: Assessing risk of bias in a non-randomized study. In: Cochrane Handbook for Systematic Reviews of Interventions.

# **“AD: Appropriate design, sampling and sample”** (Anon 2003; National Toxicology Programme 2019; Pluye et al. 2009; Sterne et al. 2020)

| **AD1:** Appropriate, scientifically based study design | “yes”: *Study design is fit for the objectives of the study; e.g., retrospective analysis of patch test results, or cross-sectional or follow-up study using a population sample*, or experimental study on relevant animal model with relevant endpoints examined | **AD1:** 1 |
| --- | --- | --- |
|  | “unclear or no” | 0 |
| **AD2:** Appropriate sample size | “adequate”: *patch test studies with at least 100 tested related to potential exposure (i.e., not patients sensitised to some index allergen such as PPD and then patch tested with other allergens, or further examined otherwise 🡪 exclusion “wrong study design”); number of animals in line with guidelines/model* | **AD2:** 1 |
|  | “partly”: *20-99 tested; number of animals not clearly reported* | 0.5 |
|  | “no”: *< 20; number of animals not reported* | 0 |
| **AD3:** Appropriate sample description | “Full”: *age (range or mean or median or anything), gender distribution, time period tested, drop-out; sex, species and strain of animals* | **AD3:** 2 |
|  | “Partial”: *any omission from above* | 1 |
|  | “No” | 0 |
| **AD4:** Selection bias | “No indication of differential selection of patients/participants into the study” *In clinical, patient-based studies, no indication of selection (other than inevitable one owing to health care system) in terms of inclusion and missing data. In epidemiological studies: appropriate sampling strategy, no indication of selective participation or loss to follow-up. In experimental studies no adequate randomization procedure, no blinding during outcome assessment if critical.* | **AD4:** 1 |
|  | “Clear indication of above” | 0 |
| **AD5:** Information bias | “No indication of exposure and/or outcome-related misclassification” *In clinical, patient-based studies, no indication of an effect of group (e.g. being hairdresser or not) on outcome reading, and vice versa. In experimental studies no information on the purity of the substance tested, no verification of the administered dose.* | **AD5:** 1 |
|  | “Clear indication of above” | 0 |
| **AD6:**Funder/sponsoring documented | “Fully/adequately”: *In studies based on routine clinical data (patient-based) institutional affiliation and a conflict of interest statement is given* | **AD6:** 1 |
|  | “Partly”: no CoI statement or no institutions given | 0.5 |
|  | “no”: both lacking | 0 |

# **“JM: Justification of methodology (validity and standards)”** (Anon 2003; National Toxicology Programme 2019; Pluye et al. 2009)

| **JM1:** Appropriate, scientifically based methodology | “Yes”: *use of patch testing to diagnose contact allergy; use of validated animal models or TG* | **JM1:** 1 |
| --- | --- | --- |
|  | “Unclear or no”: | 0 |
| **JM2:** Adequate use and description of methods | “Full”: *in patch test studies, allergen preparation (vehicle, concentration, supplier), patch test reading at least D4. In experimental studies, full conformance to OECD guideline methods/validated animal model* | **JM2:** 2 |
|  | “Partial*”: any omission from above, readings only until D3 inclusive. In experimental studies, deviations from validated/guideline methods* | 1 |
|  | “Unclear or no”: *readings only until D2 inclusive 🡪 exclusion: “wrong study design”* | 0 |
| **JM3:** Validation/standardization of used methods | “Yes”: *Patch test guidelines mentioned and followed* | **JM3:** 1 |
|  | “Partial”: *No PT guidelines mentioned, but apparently followed* | 0.5 |
|  | “No or unclear” | 0 |

# **“JR: Justification/presentation of results”** (Anon 2003; Pluye et al. 2009)

| **JR1:** Results clearly and completely documented | “Fully/adequately”: *In PT studies, number of tested and number of positives is indicated, stratified, if feasible and meaningful, for important characteristics (e.g. occupation)* | **JR1:** 1 |
| --- | --- | --- |
|  | “Partly”: *In PT studies, number of tested and number of positives can be found/derived* | 0.5 |
|  | “No or unclear” | 0 |
| **JR2:** Appropriate statistical techniques | “Fully/adequately”: *In descriptive statistics, provision of confidence intervals; when using statistical inference, appropriate statistical tests need to be employed. Analytical techniques such as multifactorial analyses need to be evaluated on a case-by-case basis* | **JR2:** 1 |
|  | “Partly”: *no confidence limits* | 0.5 |
|  | “Unclear or no”: *wrong statistical tests employed* | 0 |
| **JR3:** All measurements mentioned in the methods are reported | “Yes”: *concerning PT studies, all results with testing series/materials listed in methods are reported* | **JR3:** 1 |
|  | “No” | 0 |
| **JR4:** Independent peer review | “Yes”: if published in a peer-reviewed scientific journal | **JR4:** 1 |
|  | “Unclear or no”: otherwise | 0 |
